# Clinical Remarks upon Surgical Cases in the Buffalo General Hospital—Aneurism of the Femoral and Lower Portion of the Iliac Arteries—Ligature of the External Iliac

**Published:** 1869-02

**Authors:** J. F. Miner


ART. II.—*Clinical Remarks upon Surgical Cases in the Buffalo General Hospital—Aneurism of the Femoral and lower portion of the Iliac Arteries—Ligature of the External Iliac.* BY J. F. MINER.

GENTLEMEN:—We have this morning to present before you one of the most interesting and one of the most important forms of disease known to surgeons. The patient, John Holton, is thirty-two years of age, and in every respect of healthy constitution, so far as known. About four months since he observed the first appearance of a pulsating tumor in the left groin. It gradually increased in size and was attended by pain, both locally and extending down the leg. He now consulted physicians, who informed him of the nature of the tumor and of his great danger, advising him to visit Buffalo without delay and place himself under surgical treatment for the cure of his disease. The tumor has now attained the size of the closed hand and appears rapidly increasing in volume. He has been carefully examined by nearly all the members of the medical and surgical staff of the hospital, and no differences are expressed as to the nature of the tumor, or its termination if left uninterfered with. Various methods of treatment, however, have been proposed with the view to avoid the dangerous uncertainties and difficulties attending ligature of the external iliac, possibly of the common iliac, as it is beyond our reach to know how high the tumor may extend. In the uncertainty of this operation we all sympathize, and I undertake it with the double embarrassment of none too great confidence of success on my own part, and not any at all, on the part of nearly all my associates. It is but right under the circumstances that I state to you, and before the physicians of the city who have favored us with their presence, some of the chief reasons for making an operation so fraught with uncertainty and danger, and rejecting all other modes of procedure which surgeons have sometimes adopted successfully.

You have already been instructed as to the nature of aneurism, and understand that it is caused by the dilatation or rupture of the inner and middle coats of the artery, thus giving rise to a pulsating tumor, which is also attended by a peculiar thrill, called by the French *bruit,* which is produced by the passage of the blood current through this enlarged channel. Aneurism may be traumatic (from injury,) or idiopathic (arising from constitutional causes.) All the means of diagnosis of aneurism will be fully explained to you in the lecture-room, and it is no part of my purpose to go into details as to causes, diagnosis or treatment. I was about to explain why I had resolved to ligate the artery above this tumor rather than adopt any other expedient. Pressure can not be efficiently applied. The tumor which you observe to be irregular in outline is growing rapidly, and appears liable at any time to burst and prove immediately fatal. Though aneurism may, and occasionally does terminate in spontaneous recovery, yet such is only a rare, very rare, exception to a generally fatal termination, and in this case certainly no ground of expectation of spontaneous recovery exists. Obstruction of the vessel by coagulation of the blood or by any other means seems wholly impracticable, and we are narrowed down to the alternative of ligating the vessel and accepting the uncertainty of its results, or supinely folding our arms and waiting the natural, and, in this case, certainly fatal termination.

In no way insensible to the dangers and uncertainties of the undertaking, I propose ligating the artery above this tumor, and for the purpose of exposing the vessel proceed in the usual manner, making an incision through the integument muscles and fascia down to the peritoneum, which you now observe is carefully separated from the iliac fossa, and the external iliac artery fully exposed in its central portion. Here we place the ligature as the most safe and desirable part upon which it can be applied. Pulsation in the tumor ceases entirely upon tying the ligature. The incision, which is four and one-half inches in length, is closed by four deep and six superficial sutures and water dressings applied.

So far as the operation is concerned it has been entirely satisfactory, without accident or delay from any cause; its results, however, yet remain to be determined.* We shall watch the progress

* The patient has made satisfactory recovery. No unpleasant symptoms followed the operation; pulse at no time above 84 per minute. The moderate amount of tympanitis which was present for the first week gradually disappeared. The circulation in the leg was not manifestly disturbed, probably owing to the collateral circulation being largely established by obstruction of the blood current previous to the operation. The ligature separated from the vessel the twenty-third day, and now, thirty-six days after operation, the patient is well, except slight discharge of pus from the point where ligature rested. So far as the operation and cure of the aneurism is concerned the result is entirely successful, but the full history of the case cannot now be completed. Upon the right side, in the same place, and commencing in the same manner, is another aneurismal tumor; report of this may be expected hereafter.

of this patient with deep interest, since it is the first instance of ligature of this artery in the city, and so far as I know, in this part of the State, and since all the circumstances of the case combine to add to the natural interest which all surgeons feel in the results of their operations.

I am under many obligations to my colleagues, Drs. Rochester, Eastman and Lothrop, as well as the Hospital Physicians and Surgeons, for their efficient aid.

I am also much gratified in the interest the members of the profession have manifested in this case, and desire to express my thanks for the assistance and support I receive from the presence and encouragement of so large a number of my professional friends. If, gentlemen, I had any doubts of the propriety of my course, when I find myself sustained and encouraged by you, I know I am right.

